# Combination antibiotic therapy for community-acquired pneumonia

**DOI:** 10.1186/2110-5820-1-48

**Published:** 2011-11-23

**Authors:** Jesus Caballero, Jordi Rello

**Affiliations:** 1 Critical Care Department (VHICU), Vall d'Hebron University Hospital, Vall d'Hebron Research Institute (VHIR), Universitat Autonoma de Barcelona (UAB), Centro de Investigación Biomédica en Red de Enfermedades Respiratorias (CIBERES)Pº de la Vall d'Hebron, 119-129, 08035 Barcelona, Spain

## Abstract

Community-acquired pneumonia (CAP) is a common and potentially serious illness that is associated with morbidity and mortality. Although medical care has improved during the past decades, it is still potentially lethal. *Streptococcus pneumoniae *is the most frequent microorganism isolated. Treatment includes mandatory antibiotic therapy and organ support as needed. There are several antibiotic therapy regimens that include β-lactams or macrolides or fluoroquinolones alone or in combination. Combination antibiotic therapy achieves a better outcome compared with monotherapy and it should be given in the following subset of patients with CAP: outpatients with comorbidities and previous antibiotic therapy, nursing home patients with CAP, hospitalized patients with severe CAP, bacteremic pneumococcal CAP, presence of shock, and necessity of mechanical ventilation. Better outcome is associated with combination therapy that includes a macrolide for wide coverage of atypical pneumonia, polymicrobial pneumonia, or resistant *Streptococcus pneumoniae*. Macrolides have shown different properties other than antimicrobial activity, such as anti-inflammatory properties. Although this evidence comes from observational, most of them retrospective and nonblinded studies, the findings are consistent. Ideally, a prospective, multicenter, randomized trial should be performed to confirm these findings.

## Introduction

Community-acquired pneumonia (CAP) is a common and potentially serious illness that is associated with morbidity and mortality [1]. Only half of the cases had an etiology microorganism identified. Bacteria are the most common identifiable cause; *Streptococcus pneumoniae *is the single most common bacterium responsible. Antibiotic therapy is begun empirically, because the causative organism is not identified in a proportion of patients [1-4]. Approximately 10% of patients hospitalized with CAP are bacteremic [5]. Bacteremic *Streptococcus pneumoniae *pneumonia is the number one cause of mortality, representing up to 70% of all CAP deaths.

Although medical care has improved during the past decades, bacteremic pneumococcal pneumonia is still lethal. Explanations for this situation could be the presence of immune-compromised patients, aging population, or comorbid conditions. But there are more explanations. A severity assessment score based on the PIRO (Predisposition, Insult, Response, and Organ dysfunction) concept was assessed, including comorbidities, age greater than 70 years, multilobar opacities in chest x-ray, shock, severe hypoxemia, acute renal failure, bacteremia, and acute respiratory distress syndrome. The mean PIRO score was significantly higher in nonsurvivors than in survivors. Furthermore, analysis of variance showed that higher levels of PIRO score were significantly associated with higher mortality, prolonged length of stay in the intensive care unit (ICU), and days of mechanical ventilation. PIRO score for CAP can be used to predict 28-day mortality in CAP patients who require ICU admission [6]. Bacterial load may identify potential candidates for adjunctive therapy, ICU admission, and more aggressive management. High bacterial load appears to be significantly associated with worse outcomes [7].

Treatment of CAP continues to be a challenge in 21^st ^century. Recommendations for CAP therapy are different, depending on whether patients require hospitalization (20%) or are treated as outpatients (80%) [8]. Other issues for treatment recommendations include the emergence of antibiotic resistance among *Streptococcus pneumoniae *and mono versus combination antibiotic therapy. Combination therapy should be defined as the combination of two of the following antibiotics: β-lactam, macrolide, or fluoroquinolone. Monotherapy is the use of only one of them.

Antimicrobial therapy remains a controversial issue to treat CAP. Until better diagnostic tests are available in the clinical setting, initial treatment is usually empiric. To recommend mono versus combination therapy, we should differentiate between outpatients with CAP and patients who require hospitalization, hemodynamic support, or mechanical ventilation.

### Ambulatory setting

In Canada and the United States, macrolides remain the recommended first-line therapy for outpatients with CAP but without comorbidities. Common risk factors for CAP include age greater than 65 years, smoking, alcohol consumption, chronic lung diseases, mechanical obstruction of airways, aspiration of oropharyngeal or gastric contents, pulmonary edema, uremia, and malnutrition [9].

The recommended empirical antibiotics for outpatient therapy of CAP has been published recently in the Infectious Diseases Society of America (IDSA)/American Thoracic Society (ATS) consensus guidelines on the management of community-acquired pneumonia in adults [1]. For previously healthy patients without recent antibiotic therapy within 3 months, a macrolide or doxycycline are recommended. Macrolides remain effective for patients with mild to moderate-severe CAP with no risk factors [10]. For previously healthy patients but with antibiotic therapy within 3 months, guidelines recommend azithromycin or clarithromycin, plus a high-dose amoxicillin (4 g/day) or amoxicillin-clavulanate (4 g/day), or a respiratory fluoroquinolone alone. If there are comorbidities (chronic obstructive pulmonary disease [COPD], diabetes, renal or congestive heart failure, malignancy) but without recent antibiotic therapy, empirical therapy should start with azithromycin or clarithromycin, or a respiratory fluoroquinolone alone. If comorbidities and previous antibiotic therapy within 3 months, azithromycin or clarithromycin should be used, plus high-dose amoxicillin, amoxicillin-clavulanate, cefpodoxime, cefprozil, or cefuroxime, or a respiratory fluoroquinolone.

A respiratory fluoroquinolone is one with predictable activity against pneumococcus, such as levofloxacin or moxifloxacin. They are recommended if there is a documented allergy to β-lactams, first-line therapy fails, or against highly resistant pneumococcus prevalence. Anaerobic coverage should be given if loss of consciousness or gingival or esophageal disease. In the nursing home without hospitalization, a respiratory fluoroquinolone or amoxicillin-clavulanate plus a macrolide is recommended as the first-choice [10].

Almost all of the clinical studies comparing newer fluoroquinolones with the standard therapeutic regimen have been designed to reveal noninferiority or bioequivalence. Therefore, high-risk patients were usually excluded from these clinical trials. An outpatient trial that compared two new fluoroquinolones--gemifloxacin and trovafloxacin--showed high response rates of 92.5% and 87.3%, respectively [11]. This trial, like others, was not designed to evaluate fluoroquinolone efficacy in severe pneumonia to gain approval from registration agencies.

### CAP that requires hospitalization

Two frequent recommended antibiotic regimens for hospitalized patients with CAP are an extended-spectrum β-lactam (an extended-spectrum cephalosporin or β-lactam-β-lactamase inhibitor) with a macrolide or an antipneumococcal quinolone. These regimens have activity against the major causes of CAP, including drug-resistant *Streptococcus pneumoniae *[12-15]. Recent evidence suggests the superiority of combination therapy compared with monotherapy for subset populations, particularly patients with severe CAP, bacteremic pneumococcal CAP, or intubated CAP. Table 1 resumes all of these studies [5,16-29]. Which combination of antibiotics are more effective remains unclear, although many studies had focused on the combination of an extended-spectrum cephalosporin plus a macrolide [17,19-21,23]. Moreover, most of the studies regarding this topic are retrospective or observational, giving apparently weak scientific evidence.

**Table 1:** Published studies that favor combination therapy for in-hospital patients with CAP

Author	Year	Cohort	Site	Outcome	Study design
Gleason et al. [16]	1999	Patients aged ≥ 65 years with CAP	Ward	Lower 30-day mortality with β-lactam plus macrolide	Multicenter, retrospective

Dudas et al. [17]	2000	CAP	Ward	Lower mortality with β-lactam plus macrolide and reduced LOS	Multicenter, prospective

Waterer et al. [19]	2001	Pneumococcal bacteremia	Ward	Lower hospital mortality with combination	Multicenter, retrospective

Brown et al. [21]	2003	CAP	Ward	Lower 30-day mortality with β-lactam plus macrolide	Multicenter, retrospective

Martínez et al. [20]	2003	Pneumococcal bacteremia	Ward	Lower in-hospital mortality with β-lactam plus macrolide	Monocenter, retrospective

Baddour et al. [22]	2004	Pneumococcal bacteremia	Ward ICU	Lower 14-day mortality with combination	Multicenter, prospective

Weiss et al. [5]	2004	Pneumococcal bacteremia	Ward	Lower mortality with combination	Monocenter, retrospective

García-Vázquez et al. [23]	2005	CAP	Ward	Lower mortality with β-lactam plus macrolide	Multicenter, prospective

Mortensen et al. [24]	2006	CAP	Ward ICU	Lower 30-day mortality with β-lactam plus other than FQ	Multicenter, retrospective

Rodríguez et al. [25]	2007	CAP	ICU	Lower 28-day mortality with combination	Multicenter, retrospective

Metersky et al. [26]	2007	Pneumococcal bacteremia	Ward	Lower 30-day mortality with β-lactam plus macrolide	Multicenter, retrospective

Restrepo et al. [27]	2009	Severe sepsis pneumonia	Ward	Lower 30- and 90-day mortalities with combination plus macrolide	Multicenter, retrospective

Tessmer et al. [28]	2009	CAP	Ward	Lower 14- and 30-day mortalities with β-lactam plus macrolide	Multicenter, retrospective

Martín-Loeches et al. [29]	2010	Intubated CAP	ICU	Lower ICU mortality IDSA/ATS combination plus macrolide	Multicenter, prospective

Other studies, however, have refuted the advantages of dual therapy versus monotherapy for CAP [30-33] (Table 2). Burgess et al. [30] enrolled 213 patients treated with a nonpseudomonal third-generation cephalosporin with (116 patients) or without (97 patients) a macrolide; the majority of them (66%) received erythromycin. There were no statistical differences between patients who did or did not receive a macrolide in terms of comorbid illnesses, length of hospital stay (5.2 ± 2.8 vs. 5.2 ± 3.4 days, respectively), length of intravenous antibiotic therapy (4.4 ± 2.5 vs. 4.1 ± 2.3 days, respectively), or mortality (0.9% vs. 3.1%, respectively; *p *= 0.333). This low mortality shows that those patients did not have severe CAP. Data collected prospectively from 340 adult patients hospitalized in five countries with bacteremic pneumococcal CAP and treated with β-lactam ± macrolide were analyzed retrospectively to evaluate the efficacy of this antimicrobial combination. Univariate and multivariate analyses revealed no significant effect on case fatality rate when a macrolide/β-lactam regimen was used as initial therapy. Results were not affected by severity of illness or by excluding patients who died within 2 days of admission [31]. In a retrospective study of a cohort of 1,840 adult patients with severe sepsis or septic shock enrolled in two multicenter clinical trials between 1994 and 1999, the subanalysis of 107 patients with pneumococcal sepsis, the case-fatality rate was 20% (5/25) for patients who received antibiotic monotherapy compared with 19.5% (16/82) for patients who received combination therapy (adjusted hazard ratio [HR], 1.1; 95% confidence interval [CI], 0.4-3.1). Similarly, monotherapy did not increase the risk of death (adjusted HR, 1.0; 95% CI, 0.2-4.8) among bacteremic patients (n = 75). However, the latter analysis may have been underpowered (power, 58%) to detect a difference in mortality [32].

**Table 2:** Trials without significant difference between antibiotic monotherapy and combination therapy for CAP

Author	Year	Cohort	Outcome	Study design
Burgess and Lewiss [30]	2000	Hospitalized CAP without identified microorganism	Nonstatistical differences third-generation cephalosporin ± macrolide	Bicenter, retrospective

Dwyer et al. [31]	2006	Bacteremic pneumococcal CAP	No significant difference in case fatality if initial β-lactam + macrolide	Multicenter, retrospective

Harbarth et al. [32]	2005	Pneumococcal sepsis	Lack of effect of combo therapy	Multicenter, retrospective

Leroy et al. [33]	2005	CAP without vasopressors	Levofloxacin vs. cefotaxime + ofloxacin	Multicenter, retrospective

Monotherapy with levofloxacin (500 mg every 12 hours) was compared with combination therapy (ofloxacin 200 mg every 12 hours plus cefotaxime 1 g every 8 hours) in a multinational trial of 398 patients with CAP who required ICU admission, but who required neither vasopressors nor mechanical ventilation [33]. In this study, clinical success rates were the same for both regimens (79% vs. 80%). These results were not generalizable to intubated or patients with shock. Although the numbers were small, more patients treated with monotherapy failed a test of cure (7/29 compared with 1/27 in the combination group) [34].

In overall CAP patients, there are subsets of patients with severe CAP for whom combination therapy is clearly superior compared with monotherapy: severe CAP, bacteremic pneumococcal CAP, CAP with shock, and intubated CAP patients.

### Severe CAP

Lodise et al. demonstrated that a combination therapy of a β-lactam plus a macrolide had lower 14-day and 30-day mortality rates than with fluoroquinolone alone (14-day rates: 8.2% vs. 26.8%, *p *= 0.02; 30-days rates: 18.4% vs. 36.6%, *p *= 0.05). However, these results were shown in patients with severe CAP, defined as a class V Pneumonia Severity Index (PSI). No differences in mortality between treatment groups were noted for the lower PSI classes. The overall median length of stay (LOS) was significantly longer for the combination therapy group, but no difference in LOS was noted among PSI class V patients [35]. Severe CAP also can be defined as the presence of *Streptococcus pneumoniae *bacteremia or the necessity for aggressive ICU management due to shock, organ dysfunction, or need for mechanical ventilation.

### Bacteremic pneumococcal CAP

Recent observational studies suggest that combination therapy for severe CAP confers a significant benefit for patients, particularly those with bacteremic pneumococcal disease [5,19-22]. This group of patients represents only a small fraction of all of the CAP cases, but in terms of mortality it is highly relevant.

Monotherapy may be suboptimal for patients with severe bacteremic pneumococcal pneumonia. Waterer et al. [19] found in patients with severe bacteremic pneumococcal pneumonia a significantly higher mortality in the single effective therapy group than within the dual effective therapy group (*p *= 0.02; odds ratio [OR], 3.0; 95% CI, 1.2-7.6).

Martinez et al. [20] assessed the association between inclusion of a macrolide in a β-lactam-based empirical antibiotic regimen and mortality among patients with bacteremic pneumococcal pneumonia. Multivariate analysis showed four variables to be independently associated with death: shock (*p *< 0.0001), age ≥ 65 years (*p *= 0.02), infections with pathogens that have resistance to both penicillin and erythromycin (*p *= 0.04), and no inclusion of a macrolide in the initial antibiotic regimen (*p *= 0.03). For patients with bacteremic pneumococcal pneumonia, not adding a macrolide to a β-lactam-based initial antibiotic regimen is an independent predictor of in-hospital mortality.

Weiss et al. [5] developed a similar retrospective study on the outcome of bacteremic pneumococcal pneumonia in adults. Data showed the same results. The mortality rate was significantly higher in the group that received monotherapy with a β-lactam compared with that in the combination therapy group with a β-lactam plus a macrolide (26% vs. 7.5%, respectively; *p *= 0.02). Patients were comparable in terms of disease severity using the severity index scores).

Although all of these studies were nonrandomized, retrospective studies, they confirmed the importance of initially adding a macrolide to a β-lactam regimen for the treatment of bacteremic *Streptococcus pneumoniae *pneumonia. However, these conclusions are limited to patients with severe pneumonia for whom the mortality risk is high. The value of these results cannot be extrapolated for the treatment of inpatients with moderate disease, for which fluoroquinolones still remain an excellent therapy of choice [36].

### CAP and shock

An observational study of patients with pneumonia who require ICU care found that patients with CAP and shock who were treated with combination antibiotic therapy (58% with a third-generation cephalosporin plus a macrolide), compared with those treated with monotherapy (42% fluoroquinolone), had a higher 28-day in-ICU survival (HR, 2.69; 95% CI, 1.09-2.6). Survival was not different between combination therapy and monotherapy for ICU patients without shock [25]. As other studies, potential limitations are that it is an observational study and that the CAP severity stratification was not standardized. Although dosing was given according to 2007 IDSA/ATS guidelines [1] to define the appropriateness of empirical treatment, underdosing is a common problem in patients with severe sepsis and multiorgan dysfunction due to a higher volume of distribution [37]. The consequence is finding lower-than-expected plasma drug concentrations. However, if decreased antibiotic clearance occurs, it can lead to drug toxicity. Another limitation could be that by direct extrapolation from the 2007 IDSA/ATS CAP guidelines [1], Pseudomonas infection appears to have been suspected a great deal; more patients were chosen to receive quinolone therapy. It should not be surprising that mortality would be higher in patients suspected of possible Pseudomonas or multidrug resistant pathogens infection [38].

### Necessity of ventilator support

The effect on survival of using macrolides or fluoroquinolones in combination therapy in a cohort of intubated patients admitted to the ICU with severe CAP in 27 European ICUs was assessed [29]; 218 consecutive patients who required invasive mechanical ventilation for an admission diagnosis of CAP were recruited prospectively. Monotherapy was given in 19.7% of patients and combination therapy in 80.3%. Empirical antibiotic therapy was in accordance with the 2007 IDSA/ATS guidelines in 45.9% of patients [1]. Combination was prescribed with macrolides in 46 patients and with fluoroquinolones in 54 patients. In the macrolide group, 89.1% of patients received a third-generation cephalosporin, 4.3% a fourth-generation cephalosporin, and 6.5% received piperacillin-tazobactam. Meanwhile, 40.7% of the fluoroquinolone group received a third-generation cephalosporin as combination therapy, 22.2% received a carbapenem, and 25.9% received piperacillin-tazobactam. Mortality in the ICU was significantly lower for subjects who received a combination therapy with macrolides compared with patients who received quinolones (26.1% vs. 46.3%, *p *< 0.05). However, when excluding ciprofloxacin, no significant differences were documented. Similar results were obtained with 30-day mortality. In this cohort, a Cox regression analysis adjusted by severity identified that macrolide use was associated with lower ICU mortality (HR, 0.48; 95% CI, 0.23-0.97; *p *= 0.04) compared with the use of fluoroquinolones. When more severe patients with severe sepsis and septic shock were analyzed, similar results were obtained (HR, 0.44; 95% CI, 0.2-0.95; *p *= 0.03). Therefore, combination therapy with macrolides should be preferred for invasive mechanically ventilated patients with severe CAP.

### Factors for combination therapy

Different factors may explain the superiority of combination therapy compared with monotherapy for the treatment of patients with severe CAP. Combination therapy has a better coverage of atypical microorganisms in polymicrobial CAP, including both *Chlamydia pneumonia *and *Mycoplasma pneumonia*. It has been suggested that a proportion of bacteremic *Streptococcus pneumoniae *pneumonia patients have concomitant *Mycoplasma pneumoniae *or, rarely, Legionella sp. infections [39].

Other factor for combination therapy superiority is the fact that combination therapy acts at two different sites in the bacteria: cell wall by β-lactams, and the inhibition of protein synthesis by macrolides. Macrolides, moreover, have been shown to have some very effective anti-inflammatory properties [40]. Macrolides reduce the release of interleukin-8 and tumor necrosis factor-α, and the adherence of *Streptococcus pneumoniae *to respiratory epithelial cells. Moreover, macrolides inhibit protein synthesis, potentially decreasing the production of virulence factors [41]. Other recent studies have suggested that macrolides may have beneficial effects for severe CAP [27] because of their immunomodulatory effects rather than due to their antimicrobial properties [42]. In Berlin at the 24^th ^European Society of Intensive and Critical Care Medicine (ESICM) annual congress, Dr. Restrepo presented a retrospected analysis of the effect of macrolide therapy on 30- and 90-day mortality for patients with severe sepsis that required mechanical ventilation. After multivariable regression analysis, macrolide use in patients with severe sepsis who required intubation was associated with decreased 30-day mortality (31% vs. 53%; OR, 0.49; 95% CI, 0.41-0.6) and 90-day mortality (54% vs. 71%; OR, 0.59; 95% CI, 0.49-0.71) compared with nonmacrolide therapy after adjusting for potential confounders [43]. Confirmatory, randomized, control trials are needed to determine whether macrolide therapy may be protective for septic patients who require mechanical ventilation.

As PIRO approach defends, factors other than the simple concept of in vitro activity alone are playing key roles in bacteremic pneumonia. In fact, a lack of in vitro synergy between erythromycin and cefotaxime combined with penicillin against *Streptococcus pneumoniae *has been published [44]. Therefore, the observed effect of adding a macrolide in patients with severe CAP cannot be attributed to a synergistic action between β-lactams and macrolides [36].

## Conclusions

Combination antibiotic therapy achieves a better outcome compared with monotherapy, and it should be given in the following subset of patients with CAP: outpatients with comorbidities and previous antibiotic therapy, nursing home patients with CAP, hospitalized patients with severe CAP, bacteremic pneumococcal CAP, presence of shock, and necessity of mechanical ventilation (Table 3).

**Table 3:** Resume of recommendations for monotherapy or combination therapy in CAP

Ambulatory setting	Previously healthy patients	Monotherapy
	
	Previous antibiotic therapy	Combination or respiratory fluoroquinolone
	
	Comorbidities without previous antibiotic therapy	Monotherapy with macrolides or respiratory fluoroquinolone
	
	Comorbidities and previous antibiotic therapy	Combination therapy
**Nursing home without hospitalization**	CAP	Combination therapy

**Hospitalized CAP**	Moderate disease	Monotherapy with respiratory fluoroquinolones or combination therapy
	
	Severe CAP	Combination therapy
	
	Bacteremic pneumococcal CAP	Combination therapy
	
	CAP and shock	Combination therapy
	
	Ventilation support	Combination therapy

At ambulatory setting, if comorbidities and previous antibiotic therapy within 3 months, azithromycin or clarithromycin should be used, plus high-dose amoxicillin, amoxicillin-clavulanate, cefpodoxime, cefprozil, or cefuroxime, or a respiratory fluoroquinolone. In previously healthy patients without comorbidities or without previous antibiotic therapy, monotherapy is still the empirical initial recommended therapy. In the nursing home without hospitalization, a respiratory fluoroquinolone or amoxicillin-clavulanate plus a macrolide is recommended as the first choice.

Initial empirical combination therapy of a cephalosporin plus a macrolide for patients with CAP who require hospitalization is associated with decreased mortality and or shorter hospital stay than treatment with a cephalosporin alone. For patients with moderate disease, fluoroquinolones remain an excellent therapy of choice. Although this evidence comes from observational, retrospective, and nonblinded studies, the findings are consistent. For practical purposes, for the majority of patients with CAP in ambulatory settings or hospitalized and not severely ill, fluoroquinolone monotherapy remains an approved, tested, cost-effective, and reliable option. Otherwise, hospitalized patients with a severe CAP, bacteremia, ICU admission, or need for vasopressors or mechanical ventilation may benefit from a dual antibiotic therapy that combines a third-generation cephalosporin and a macrolide. Ideally, a prospective, randomized trial should be performed to confirm these findings, although severe heterogenicity of patients is a challenge to enroll patients and find differences as several sepsis trials show.

## Competing interests

Jesus Caballero declares that he has no competing interests. Jordi Rello declares that he serves on advisory boards and speakers' board for Pfizer and Novartis.

## Authors' information

JR is a well-known expert in critical infectious diseases and chief of Vall d'Hebron Critical Care Department. He has a wide background on pneumonia, either hospital or community-acquired. JC is an attending physician of Vall d'Hebron Critical Care Department.

## Authors' contributions

JC wrote the first draft of the manuscript. JR revised the manuscript. Both authors read and approved the final manuscript.
